# Flavonoids and Omega3 Prevent Muscle and Cardiac Damage in Duchenne Muscular Dystrophy Animal Model

**DOI:** 10.3390/cells10112917

**Published:** 2021-10-28

**Authors:** Luana Tripodi, Davide Molinaro, Andrea Farini, Gendenver Cadiao, Chiara Villa, Yvan Torrente

**Affiliations:** Stem Cell Laboratory, Department of Pathophysiology and Transplantation, Università degli Studi di Milano, Centro Dino Ferrari, Fondazione IRCCS Cà Granda Ospedale Maggiore Policlinico, 20122 Milan, Italy; luana.tripodi@unimi.it (L.T.); molinarodavide97@yahoo.com (D.M.); farini.andrea@gmail.com (A.F.); g.cadiao@gmail.com (G.C.)

**Keywords:** muscle homeostasis, muscle regeneration, satellite cells, inflammatory response, Duchenne muscular dystrophy, food supplement

## Abstract

Nutraceutical products possess various anti-inflammatory, antiarrhythmic, cardiotonic, and antioxidant pharmacological activities that could be useful in preventing oxidative damage, mainly induced by reactive oxygen species. Previously published data showed that a mixture of polyphenols and polyunsaturated fatty acids, mediate an antioxidative response in mdx mice, Duchenne muscular dystrophy animal model. Dystrophic muscles are characterized by low regenerative capacity, fibrosis, fiber necrosis, inflammatory process, altered autophagic flux and inadequate anti-oxidant response. FLAVOmega β is a mixture of flavonoids and docosahexaenoic acid. In this study, we evaluated the role of these supplements in the amelioration of the pathological phenotype in dystrophic mice through in vitro and in vivo assays. FLAVOmega β reduced inflammation and fibrosis, dampened reactive oxygen species production, and induced an oxidative metabolic switch of myofibers, with consequent increase of mitochondrial activity, vascularization, and fatigue resistance. Therefore, we propose FLAVOmega β as food supplement suitable for preventing muscle weakness, delaying inflammatory milieu, and sustaining physical health in patients affected from DMD.

## 1. Introduction

Dietary supplementation is an important source of vitamins, minerals, herbs, or products made from plants, animals, algae, seafood, or yeasts, intended to complement the common diet in physiological or pathological conditions. In fact, food supplements may be useful for the maintenance of a proper nutrient supply to promote health span and improve physical performance of healthy subjects, in case of nutritional deficiencies and as an adjuvant tool for the management of multiple disease conditions.

Neuromuscular disorders, such as Duchenne muscular dystrophy (DMD), present important secondary pathologic features that fulfil the requirements for dietary supplementation. DMD is an X-linked recessive disease that affects muscular function and strength. It is caused by mutations of the dystrophin gene, which result in null expression of the structural protein, leading to instability of the dystrophin-associated glycoprotein complex and sarcolemma fragility. DMD muscles are characterized by low regenerative capacity, fibrosis, fiber necrosis [1], inflammatory infiltrates, intracellular Ca^2+^ dysregulation, aberrant cellular signaling, mitochondrial malfunction and overproduction of reactive oxygen species (ROS), which outweigh a physiological antioxidant response [2]. Heart complications and respiratory disorders are manifested as the pathology progresses and are the major causes of death in DMD patients [3]. Among cardiac complications, dilated cardiomyopathy (DCM) leads to increased ventricular chamber size, coupled with loss of contractile function (ejection fraction < 40%) [4], progressive myocardial fibrosis and decreased cardiac function [5].

FLAVOMEGA, a dietary supplementation targeting inflammatory response and oxidative stress, and consisting in a mixture of omega-3 fatty acids (O-3FAs), phospholipidic curcumin, acetyl carnitine-l-HCL, coenzyme Q10 (CoQ10), Scutellaria baicalensis Georgi baicalein, Green Tea (Camellia sinensis) epicatechins, and other micronutrients, had already been tested by our group in DMD treatment, both in murine animal models and in clinical trial enrolling patients with DMD [6,7].

We have recently developed FLAVOmega β, an improved version of FLAVOMEGA. We selected the compounds with the highest beneficial role among those of FLAVOMEGA, namely O-3FAs, phospholipidic curcumin, CoQ10, baicalein, green tea epicatechins, and added aloe vera. Moreover, the dose of each component was adjusted. In the literature, these supplements have already been described and proposed as a dietary treatment that regulates the level of metabolites, decreases free radicals, activates antioxidant enzymes, and modulates mitochondrial respiratory chain function with high energy production [8,9,10].

Curcumin is known for its antioxidant and anti-inflammatory properties. It acts as a scavenger against ROS, favors the neutralization of oxidants by endogenous enzymes (glutathione peroxidase, catalase, superoxide dismutase) and inhibits ROS-producing ones (lipoxygenase, cyclooxygenase and xanthine hydrogenase/oxidase) [11]. The anti-inflammatory role of curcumin is explicated by the reduction of serum concentrations of TNF- α and other inflammatory cytokines, and the modulation of the NF-κB pathway [12]. Administration of supplements enriched in curcumin improves endurance and muscular performance by reducing muscle fiber necrosis and fibrosis deposition, and by increasing muscle mass [6].

CoQ10 is an excellent electron carrier able to support continuous oxidation-reduction cycles [13]. It alleviates aging symptoms and cardiovascular diseases via its antioxidant activity [14]. Moreover, CoQ10 administration reduces levels of circulating C-reactive protein, TNF-α and IL-6 in pathologies with an inflammatory background, such as cardio-cerebral vascular diseases, multiple sclerosis, obesity, renal failure, rheumatoid arthritis, diabetes, and fatty liver disease [15].

Similar to the above-mentioned compounds, the flavonoid baicalein is a scavenger for ROS and a modulator of multiple enzymes involved in inflammatory processes (nitric oxide synthase, cyclooxygenases, lipoxygenases), cellular adhesion molecules and chemokines [16]. In addition, baicalein performs a protective function against death receptor-dependent necrosis in cardiomyocytes, as it prevents necrosome formation mediated by receptor interacting serine/threonine kinases 1 and 3 (RIPK1/RIPK3) [17].

Finally, aloe vera is rich in vitamins, minerals, sugars, amino acids, fatty acids and hormones, and possesses multiple beneficial properties, ranging from antibacterial and antiviral activities to analgesic, anti-inflammatory and antioxidant effects [18].

Despite the continuous therapeutic innovation through several clinical trials, an effective treatment for DMD pathology does not exist. However, food supplement administration constitutes a promising approach to support the management of DMD symptoms. In this work, we demonstrate the favorable role of FLAVOmega β in the treatment of mdx animal model. Our analysis showed a potential role of FLAVOmega β in stimulating proliferation, myogenicity, vascular remodeling and an oxidative metabolic switch of myofibers. Based on our results, we propose its use as a food supplement to improve the quality of life and the pathological phenotype of DMD patients.

## 2. Materials and Methods

### 2.1. Animal Statement

All procedures involving living animals were performed in accordance with Italian law (D.L.vo 116/92 and subsequent additions), which conforms to the European Union guidelines. The use of animals in this study was authorized by the National Ministry of Health (protocol number 10/13–2014/2015). Eight mdx mice (C57BL6/10ScSn-DMDmdx/J) 3 months of age were provided by Charles River. All animals were housed in a controlled environment (12 h light/dark cycle) at a temperature between 21 and 23 °C. Cage population was limited to a maximum of four animals each to ensure the health and welfare of animals. Mice had free access to clean water and food. Systemic administration of 100 μL of FLAVOmega β (100 μg/μL) per mouse was performed by oral gavage for 4 weeks (1 oral administration per day). Untreated aged-matched mdx mice were used as controls. After treatment, mice were deeply anesthetized and then sacrificed by cervical dislocation.

### 2.2. Supplement Formulations

Six different formulations of the supplement mixture were produced, as shown in Table 1. FLAVO α, FLAVO β, FLAVO γ only consist of a powder phase of curcumin, CoQ10, Scutellaria dry extract (baicalein), green tea extract (epicatechins) and aloe vera, at variable concentrations (1X, 5X and 10X, respectively). An oil phase with variable concentrations of Docosahexaenoic acid (DHA) and Eicosapentaenoic acid (EPA) (1X, 5X and 10X, respectively) was added to FLAVO α to obtain FLAVOmega α, FLAVOmega β and FLAVOmega γ. All components were bought as pure and directly dissolved in 0.1% dimethyl sulfoxide (DMSO). DMSO is a class III solvent according to FDA classification (International Council for Harmonisation of Technical Requirements for Pharmaceuticals for Human Use (ICH) guidance for industry Q3C Impurities: Residual Solvents). 0.1% is the lowest concentration of DMSO that allows complete dissolution of all components in absence of cytotoxic effects. Preliminary studies confirmed that there is no difference in cytotoxicity between normal growth medium, 0.01% and 0.1% DMSO.

### 2.3. Optical Properties of FLAVOmega β

Photoluminescence (PL) and time-resolved PL spectra were recorded with a Cary Varian Eclipse at a 90°-degree incidence, with a bandpass of 5 nm. Absorption spectra were recorded with a Cary Varian 50 spectrophotometer using Quartz Suprasil cuvettes with optical path of 1 cm at normal incidence with a band pass of 0.5 nm. Ethanol (EtOH) and ultrapure water (H_2_O) were used as solvents.

### 2.4. MTT-Based Cell Proliferation Assay

3-(4,5-Dimethylthiazol-2-yl)-2,5-Diphenyltetrazolium Bromide (MTT)-based cell proliferation assay was performed for the following conditions: untreated C2C12 cells (CTRL), C2C12 cells treated with vehicle of the mixture (CTRL DMSO), C2C12 treated with formulation FLAVO α, FLAVO β, FLAVO γ, FLAVOmega α, FLAVOmega β and FLAVOmega γ. CTRL, CTRL DMSO and FLAVOmega β were also tested in conditions of high oxidative stress, via addition of H_2_O_2_ (250 µM) to the medium. A total of 5.000 cells/well (*n* = 3 wells per condition) were seeded in 96-well plates and MTT test was performed at different time points after treatment (0–8–24–48–72–96 h). MTT labelling reagent was added to cell culture media at 1:10 (*v*/*v*) dilution and incubated for 4 h at 37 °C and 5% of CO_2_. MTT was then aspirated from each well and 100 uL of DMSO were added for solubilization. Absorbance was measured by means of GloMax Discover System (Promega Corporation, Madison, WI, USA) at 570 nm.

### 2.5. ROS Detection

ROS production was evaluated for the following conditions: CTRL, CTRL DMSO, C2C12 treated with formulation FLAVO α, FLAVO β, FLAVO γ, FLAVOmega α, FLAVOmega β and FLAVOmega γ. CTRL, CTRL DMSO and FLAVOmega β were also tested in conditions of high oxidative stress, via addition of H_2_O_2_ (250 µM) to the medium. A total of 5.000 cells/well (*n* = 3 wells per condition) were seeded into a 96-well plate, as described for MTT test. The analysis was performed at 8–24–48–72–96 h after treatment by means of ROS-Glo™ H_2_O_2_ Assay (Promega Corporation, Madison, WI, USA) lytic procedure, following manufacturer’s instructions. Relative luminescence units were measured by GloMax Discover System (Promega Corporation, Madison, WI, USA) at 570 nm.

### 2.6. Fusion Index and Desmin Immunofluorescence Staining

C2C12 cells at passage 5 were seeded at a density of 20.000 cells/well (*n* = 3 wells per condition) and grown in DMEM supplemented with 15% Fetal Bovine Serum (FBS) and Penicillin/Streptomycin. Horse serum (HS) (2%) is normally added to the growth medium when a confluence of 80% is reached, in order to induce differentiation into myotubes. Three growth conditions were then set: culture in DMEM with 15% FBS (growth medium), in DMEM with 15% FBS and 2% HS (diff. medium) and in DMEM with 15% FBS and 2% HS plus FLAVOmega β (diff. medium + FLAVOmega β). For FLAVOmega β administration, a 2.7 mg/µL formulation was produced and diluted 1:27 (*v*/*v*) in growth medium. Fusion index was calculated after 7 days of culture. Cells were fixed with methanol-free 4% paraformaldehyde (PFA) (Thermo Fisher Scientific, Waltham, MA, USA) for 10 min at room temperature (RT), followed by 3 washings in phosphate-buffered solution (PBS) 1X, 5 min each. Then, cells were incubated with a blocking solution consisting of 5% FBS and 2% HS in PBS 1X at RT for 30 min, followed by incubation with anti-desmin primary antibody (1:100, ab15200, Abcam, Cambridge, UK) diluted in blocking solution for 90 min at RT. After 3 PBS 1X washes of 5 min, cells were incubated with goat anti-rabbit 488 secondary antibody diluted 1:100 in PBS 1X for 1 h. Nuclei were counterstained with 4,6-diamidino-2-phenylindole (DAPI) (Molecular Probes, Thermo Fisher Scientific, Waltham, MA, USA) for 5 min at RT. Slides were mounted with PBS 1X-Glycerol (Sigma-Aldrich, St. Louis, MO, USA) at a 1:1 ratio and with coverslips. A Leica DMi8 fluorescence microscope was used for acquiring the images. Fusion index was calculated as the ratio between the number of nuclei located on desmin-positive signal and the total number of nuclei per section.

### 2.7. Assessing Functional Performance

Functional performance of mdx mice was evaluated by Rotarod running test to measure balance and motor coordination; treadmill test was performed to evaluate fatigue resistance.

### 2.8. Histological Analysis

Muscles and hearts were collected from mdx mice treated with 100 μL FLAVOmega β and untreated mice, frozen in liquid hydrogen-cooled isopentane, and cut by cryostat into 8 µm sections. Azan Mallory (AM) and Hematoxylin and Eosin (H&E) staining were performed as described in [19].

### 2.9. Immunofluorescence Analysis

Sections were brought at RT from −80 °C and fixed with 4% PFA (Thermo Fisher Scientific, Waltham, MA, USA) for 10 min at RT, followed by 3 washings in PBS 1X, 5 min each. PBS 1X + 0.01% Triton X-100 (Sigma-Aldrich, St. Louis, MO, USA) for 20 min at RT was used to permeabilize tissues. Sections were then incubated with a blocking solution of 10% donkey serum in PBS 1X, for 1 h at RT. Sections were then incubated overnight at 4 °C with rat anti-mouse CD31 (1:50, Clone MEC 13.3, BD PharMingen, Franklin Lakes, NJ, USA) primary antibody, diluted in blocking solution (1:50). After 3 PBS 1X washes of 5 min, donkey anti-rat secondary antibody Dylight 650 (1:100, SA5-10029, Invitrogen Thermo Fisher Scientific, Waltham, MA, USA) was diluted in PBS 1X and added for 1h at room temperature to sections. After PBS 1X washing, fluorescent conjugated Isolectin-594 (1:100, I21413, Invitrogen Thermo Fisher Scientific, Waltham, MA, USA) and α-SMA Fitc (1:100, F3777, Sigma-Aldrich, St. Louis, MO, USA) antibodies diluted in PBS were incubated for 1h at RT. Nuclei were counterstained with DAPI (Molecular Probes, Thermo Fisher Scientific, Waltham, MA, USA) for 5 min at RT. Slides were mounted with PBS 1X-Glycerol (Sigma-Aldrich, St. Louis, MO, USA) at 1:1 ratio and coverslips. Images were acquired with a Leica DMi8 fluorescence microscope.

### 2.10. Muscle Fiber Type Immunofluorescence

Sections were fixed with ethanol and acetone solution diluted 1:1 for 3 min at RT, followed by 3 washings in PBS 1X, 5 min each. Antigen retrieval was performed by immersion in sodium citrate buffer (10mM) at pH 6 at 100° for 25 min, followed by a 15 min cooling period and 3 washings with 0.05% Tween in PBS1X, 3 min each. Sections were incubated for 1 h with a blocking solution of 5% goat serum in PBS 1X at RT, and then incubated overnight at 4 °C with primary antibody, diluted in blocking solution: Myosin heavy chain Type I BA-D5 (1:50, 115-675-207, DSHB, Iowa City, Iowa, IA, USA), Myosin heavy chain Type IIa sc-71 (1:50, 115-545-205, DSHB, Iowa City, Iowa, IA, USA), Myosin heavy chain Type IIb BF-F3 (1:50, 115-585-075, DSHB, Iowa City, Iowa, IA, USA). After 3 PBS 1X washes of 5 min, goat anti-mouse IgG 488 was diluted 1:100 in PBS 1X and added for 40 min at 37 °C to sections. After PBS 1X washing, goat anti-mouse IgG 421 and goat anti-mouse IgM 594 were diluted 1:100 in PBS 1X and added for 40 min at 37 °C to sections. Sections were washed with PBS 1X and incubated with laminin primary antibody (1:200, L9393, Sigma-Aldrich, St. Louis, MO, USA) at RT for 2 h. After PBS 1X washing, goat anti-rabbit IgG 647 was diluted 1:100 in PBS 1X and added at 37 °C for 40 min to sections. Slides were mounted with PBS 1X-Glycerol (Sigma-Aldrich, St. Louis, MO, USA) at 1:1 ratio and coverslips. A Leica DMi8 fluorescence microscope was used for acquiring images.

### 2.11. Immunohistochemistry Staining

Endogenous peroxidase activity of tissue slices was blocked with 0.3% alcoholic hydrogen peroxide for 30 min at RT. After PBS 1X washing, antigen retrieval was performed in sodium citrate buffer [10 mM] pH 6 for 30 min at 100 °C, followed by a 15 min cooling period and 3 washings with 0.05% Tween in PBS 1X for 3 min each. Sections were incubated with a blocking solution of 5% HS and 5% FBS in PBS 1X for 1 h at RT, and then incubated with PAX7 (1:50, AB92317, Abcam, Cambridge, UK) primary antibody, diluted 1:20 in blocking solution, ON at 4 °C. After PBS 1X washing, sections were incubated with appropriate biotinylated immunoglobulin antibodies for 30 min at RT, followed by incubation with peroxidase–avidin–biotin complex (Vectastain ABC Elite kit; Vector Labs, Burlingame, CA, USA) for 30 min. 3,30-Diaminobenzidine (DAB) was used as the chromogen. Eosin 1% diluted 1:50 in dH_2_O solution was used to stain myofibers for 30 s. Dehydration was performed by immersing slides into increasing ethanol dilutions in water (Carlo Erba, Milan, Italy), for 45–60 s. 100% xylene (Sigma-Aldrich, St. Louis, MO, USA) and DPX (VWR International, Radnor, PA, USA) were used to mount slides with coverslips. Acquisition of section images was carried out using a laser microdissection microscope (LMD6000B, Leica Microsystems, Wetzlar, Germany).

### 2.12. Western Blot Analysis

Skeletal muscles and cardiac tissues were isolated from treated and untreated 3m mdx mice and total proteins were obtained as in [20]. Briefly, tibialis anterior muscles and hearts were homogenized and resuspended with NP40 buffer with Phostop and Complete protease inhibitors 10X. After 30 min at 4 °C, lysates were centrifuged at 13.000 rpm for 10 min. Supernatants were stored at −80 °C and the pellets discarded. Protein concentration was determined by Bradford Assay absorbance with a Promega™ GloMax^®^ system and software. For Western blot analysis, samples were resolved on polyacrylamide gels (ranging from 6% to 14%) and transferred to nitrocellulose membranes (Bio-Rad Laboratories, Hercules, CA, USA). Filters were incubated overnight with the following antibodies: LC3B (1:500, L7543, Sigma-Aldrich, St. Louis, MO, USA); TNF-α (1:500, e-ab-40015, Elabscience, Wuhan, China); PAX7 (1:500, AB92317, Abcam, Cambridge, UK); MYOD1 (1:500, M3512, DAKO, Glostrup, Denmark); PTX3 (1:500, AB90806, Abcam, Cambridge, UK); IL-6 (1:500, sc-57315, Santa Cruz Biotechnology, Dallas, TX, USA); TLR2 (1500, orb229137, Biorbyt, St. Louis, MO, USA): TLR4 (1:500, sc-293072, Santa Cruz Biotechnology, Dallas, TX, USA); Vinculin (1:500, MA5-11690, Invitrogen Thermo Fisher Scientific, Waltham, MA, USA); MMP9 (1:500, ab38898, Abcam, Cambridge, UK); ATG7 (1:500, sab4200304, Sigma-Aldrich, St. Louis, MO, USA); SMAD3 (1:500, e-ab-32921, Elabscience, Wuhan, China); P62 (1:500, P0067, Sigma-Aldrich, St. Louis, MO, USA); phosphoSMAD2-3 (1:500, E-AB-21-040, Elabscience, Wuhan, China); RAGE (1:500, NBP2-03950, NOVUSBIO, Centennial, CO, USA); S-100β (1:500, sc-393919, Santa Cruz Biotechnology, Dallas, TX, USA); OXPHOS complex (1:500, ab110413, Abcam, Cambridge, UK); eNOS (1:500, ab76198, Abcam, Cambridge, UK); PGC-1α (D-5, 1:500, sc-518025, Santa Cruz Biotechnology, Dallas, TX, USA). Proteins were detected with peroxidase conjugated secondary antibodies (Agilent Technologies, Santa Clara, CA, USA) and developed by ECL (Amersham Biosciences, Amersham, UK). The following exposition process was carried out in the ChemiDoc Imaging System (Bio-Rad Laboratories, Hercules, CA, USA).

### 2.13. Image Quantification and Statistical Analysis

Quantitative analyses of H&E, AM, immunochemistry and immunofluorescence staining were performed by ImageJ Software (NIH). A threshold color plug-in of ImageJ Software was used to quantify AM staining as percentage of area over a fixed grid area. Data were analyzed using GraphPad Prism^TM^ and expressed as means ±SD. To compare multiple group’s means, one-way ANOVA and two-way ANOVA followed by Tukey’s multiple comparison test were used to determine significance (* *p* < 0.05, ** *p* < 0.01, *** *p* < 0.001; **** *p* < 0.0001). To compare two groups, Student’s *t*-test was applied assuming equal variances (* *p* < 0.05, ** *p* < 0.01, *** *p* < 0.001; **** *p* < 0.0001).

## 3. Results

**Figure 1:**
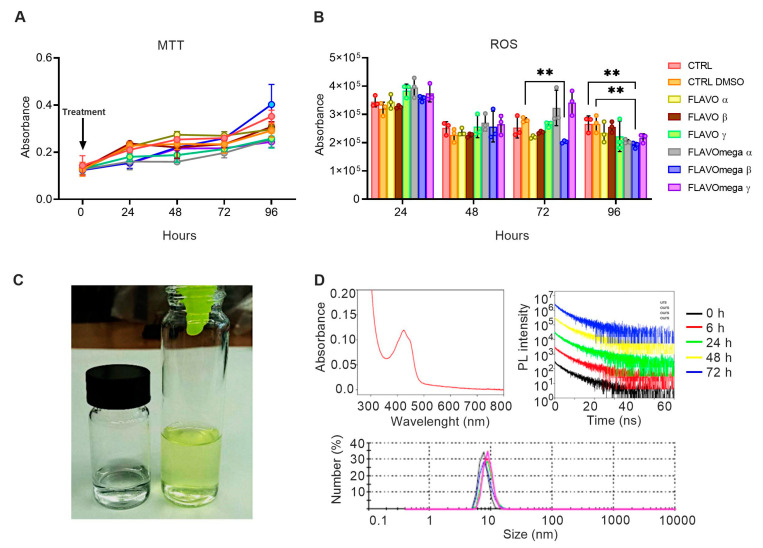
(**A**,**B**) MTT-based cell proliferation assay and ROS detection assay (0–24–48–72–96h) on untreated C2C12 cells (CTRL), C2C12 cells treated with vehicle of FLAVOmega β (CTRL DMSO), C2C12 treated with formulation FLAVO α, FLAVO β, FLAVO γ, FLAVOmega α, FLAVOmega β and FLAVOmega γ (*n* = 3 for each experimental group). Relative luminescence units were measured by GloMax Discover System (Promega Corporation, USA) at 570 nm. (**B**) Two-way ANOVA followed by Tukey’s multiple comparison test: C2C12 cells treated with FLAVOmega β compared to CTRL DMSO (** *p* = 0.0040) at 72 h after treatment; C2C12 treated with FLAVOmega β compared to CTRL (** *p* = 0.0048) and CTRL DMSO (** *p* = 0.0046) at 96 h. All values are expressed as the mean ± SD. (**C**) Representative image of FLAVOmega β formulation. (**D**) Absorption spectrum of FLAVOmega β formulation (top left); time-resolved PL of FLAVOmega β formulation (top right); dynamic light scattering (DLS) analysis of FLAVOmega β formulation (bottom).

We synthetized six different formulations of our mixture (FLAVO α, FLAVO β, FLAVO γ, FLAVOmega α, FLAVOmega β, FLAVOmega γ) and performed preliminary assays on C2C12 cell cultures to evaluate their effects on proliferation and antioxidant activity, and to select the formulation with the best dose-response trait. MTT-based cell proliferation assay showed slightly increased absorbance through time, but the viability was not significantly modified (Figure 1A). ROS detection showed a decrease of reactive oxygen species production in cell culture medium after treatment. In particular, we observed a significant decrease of ROS production in C2C12 cells treated with FLAVOmega β compared to CTRL DMSO (*p* = 0.0040) at 72 h after treatment, and in C2C12 treated with FLAVOmega β compared to CTRL (*p* = 0.0048) and CTRL DMSO (*p* = 0.0046) at 96 h (Figure 1B). It should be noted that the overall decrease in ROS production observed at 48 h compared to 24 h, for all experimental groups, could be attributed to the adaptation of the cell population to the culture conditions following splitting [21]. Based on these preliminary results, we selected FLAVOmega β for further evaluations.

### 3.1. Physical Properties of FLAVOmega β

Macroscopically, FLAVOmega β appears as a clear, yellow-green solution, compared to the vehicle solution, which is colorless (Figure 1C). The absorption spectrum of the mixture shows a peak at approximately 425 nm, which corresponds to the one of curcumin [22]. Dynamic Light Scattering (DLS) analysis demonstrates that the synthesized mixture is a homogenous emulsion composed of particles with an average size of 10 nm. To assess the stability of FLAVOmega β we recorded the time-resolved PL spectra at 0, 6, 24, 48 and 72 h and observed no signs of particle degradation for the whole duration of the analysis, as demonstrated by the unchanged emission decay at different time points (Figure 1D).

### 3.2. FLAVOmega β Increases Proliferation and Myogenicity of C2C12 Myoblasts

**Figure 2:**
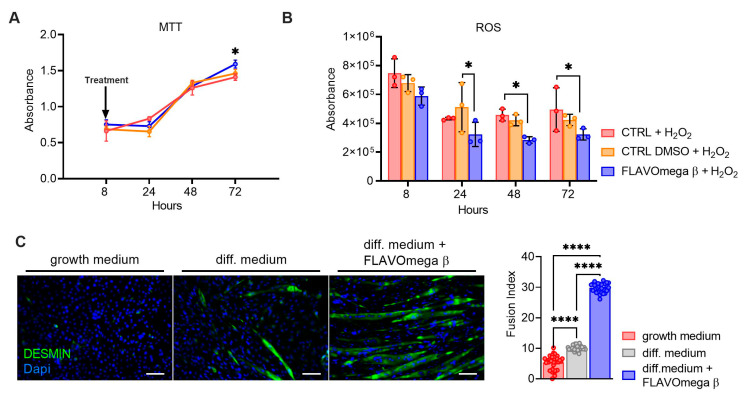
(**A**,**B**) MTT-based cell proliferation assay and ROS detection assay (8–24–48–72 h) on CTRL, CTRL DMSO and C2C12 treated with FLAVOmega β formulation (*n* = 3 for each experimental group) with addition of H_2_O_2_. Relative luminescence units were measured by GloMax Discover System (Promega Corporation, USA) at 570 nm. (**A**) MTT-based cell proliferation assay: Two-way ANOVA: C2C12 treated with FLAVOmega β compared to CTRL at 72 h (* *p* = 0.0288) after treatment. (**B**) ROS detection assay: Two-way ANOVA: C2C12 treated with FLAVOmega β compared to CTRL DMSO at 24 h (* *p* = 0.0273); C2C12 treated with FLAVOmega β compared to CTRL at 48 h (* *p* = 0.0450) and 72 h (* *p* = 0.0460) after treatment. All values are expressed as the mean ±SD. (**C**) Immunofluorescence analysis of desmin (green) and DAPI (blue) in C2C12 cells with growth medium, C2C12 with differentiation medium and C2C12 with differentiation medium treated with formulation FLAVOmega β. Scale bar: 200 µm. In the lateral panel, comparison of the calculated fusion index for the three conditions. *t*-test: (**** *p* < 0.0001).

MTT-based cell proliferation and ROS detection assays were repeated in conditions of high oxidative stress for CTRL, CTRL DMSO and FLAVOmega β groups. In MTT experiment we observed increased cell proliferation in C2C12 treated with FLAVOmega β (*p* = 0.0288) compared to CTRL at 72 h after treatment (Figure 2A). Evaluation of ROS production in cell culture media showed an important effect of FLAVOmega β starting from 24 h after treatment; in particular, we observed a ROS decrease in C2C12 treated with FLAVOmega β compared to CTRL DMSO at 24 h (*p* = 0.0273). In addition, we observed a ROS production decrease in C2C12 treated with FLAVOmega β compared to CTRL at 48 h (*p* = 0.0450) and at 72 h (*p* = 0.0460) after treatment (Figure 2B). To assess whether FLAVOmega β can actually influence muscle homeostasis, we evaluated the differentiation capacity of C2C12 cells after FLAVOmega β treatment. In presence of FLAVOmega β, C2C12 cells showed a higher myogenic capacity, as confirmed by the increased number of desmin positive myotubes, compared to control C2C12 cultured in differentiation medium (*p* < 0.0001) (Figure 2C).

### 3.3. Treatment with FLAVOmega β Improves Skeletal Muscle Performance and Dystrophic Phenotype

**Figure 3:**
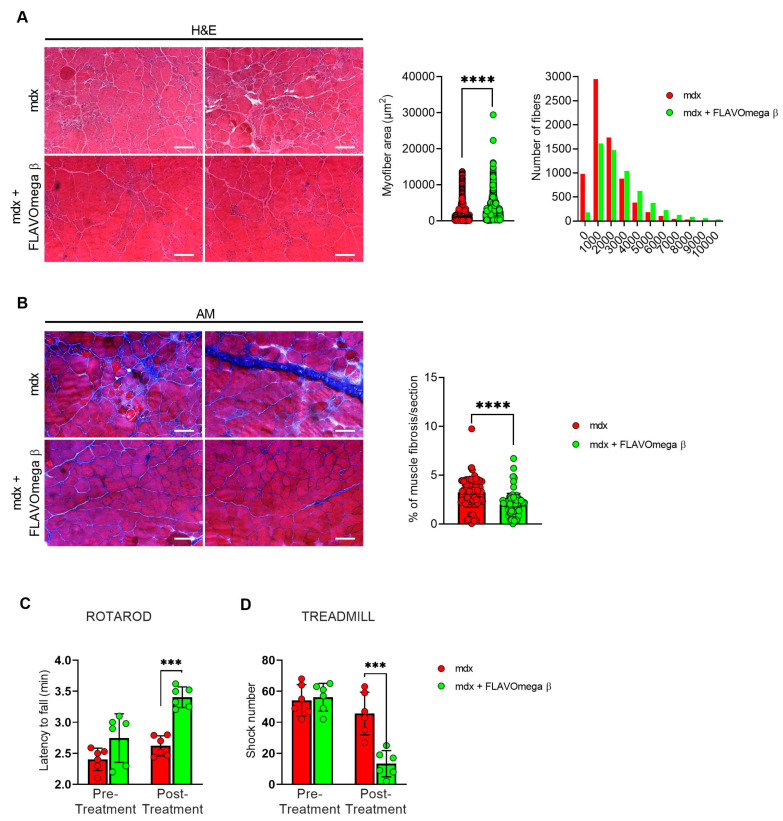
(**A**) Representative images of H&E staining of mdx mice treated with FLAVOmega β and untreated mdx mice skeletal muscles (*n* = 3 mice; *n* = 1497 slices/3m mdx, *n* = 5883 slices/3m mdx + FLAVOmega β). Scale bar: 200 mm. Quantification of the relative frequency, expressed as the frequency distribution, of cross-sectional areas (CSA) of myofibers in TA of mdx mice treated with FLAVOmega β and untreated mdx mice. Boxes indicate 25th to 75th percentiles. *t*-test: **** *p* < 0.0001. (**B**) Representative AM staining images of mdx mice treated with FLAVOmega β and untreated mdx mice skeletal muscles (*n* = 3 mice; *n* = 186 slices/3m mdx, *n* = 109 slices/3m mdx + FLAVOmega β. Histogram represents the percentage of fibrotic area per section of mdx mice. Scale bar: 200 µm. *t*-test: *** *p* < 0.0001. (**C**,**D**) Evaluation of coordination, balance and fatigue resistance with the Rotarod running test and the treadmill exercise of mdx mice treated with FLAVOmega β and untreated mdx mice (*n* = 6 mice/3m mdx, *n* = 6 mice/3m mdx + FLAVOmega β). (**C**) Histogram represents the average latency of the 3-month-old mdx mice treated with FLAVOmega β and age-matched mdx controls. *t*-test: *** *p* = 0.0001. (**D**) Histogram represents the number of shocks suffered by 3-month-old mdx mice treated with FLAVOmega β and age-matched mdx controls. *t*-test: *** *p* = 0.0002. All values are expressed as the mean ± SD.

**Figure 4:**
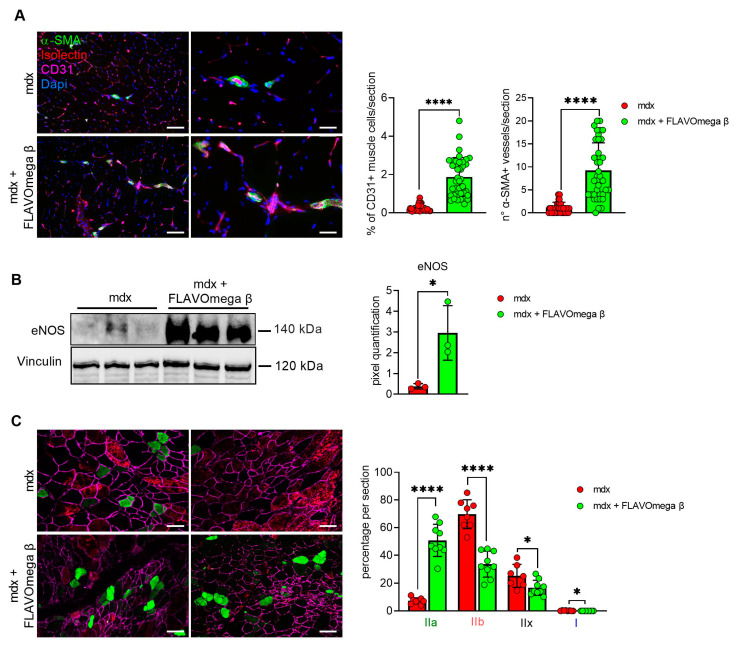
(**A**) Immunofluorescence analysis of CD31 (violet), isolectin (red) and α-SMA (green) in skeletal muscles of mdx mice treated with FLAVOmega β and untreated mdx mice (*n* = 3 mice/each group, *n* = 9 sections/3m mdx, *n* = 9 sections/3m mdx + FLAVOmega β). Nuclei were counterstained with DAPI (blue) Scale bar left panel: 100 µm; scale bar right panel: 25 µm. *t*-test: **** *p* < 0.0001. All values are expressed as mean ± SD. (**B**) Western blot (WB) analysis of eNOS protein in skeletal muscles of mdx mice treated with FLAVOmega β and untreated mdx mice (*n* = 3 mice/each group). In the lateral panel, densitometric analysis of data, expressed as the ratio of different proteins on vinculin in arbitrary units. *t*-test: * *p* = 0.0282. (**C**) Immunofluorescence analysis of type IIa (green), IIb (red), IIx (black), I (blue) fibers in skeletal muscles of mdx mice treated with FLAVOmega β and untreated mdx mice (*n* = 3 mice/each group, *n* = 7–8 slices/3m mdx, *n* = 9 slices/3m mdx + FLAVOmega β). Scale bar: 100 µm. *t*-test: **** *p* < 0.0001, * *p* = 0.0252, * *p* = 0.0134. All values are expressed as mean ± SD.

We performed histological analysis in 3-month-old dystrophic mice treated with FLAVOmega β, compared to age-matched mdx, to evaluate fiber morphology and fibrotic deposition. Three-month-old dystrophic mice treated with FLAVOmega β displayed an increased myofiber area (*p* < 0.0001) compared to age-matched untreated mdx mice. The values of frequency distribution confirmed large area of myofibers in mice that received FLAVOmega β administration (25% Percentile 3m mdx: 1488, 8285; 25% Percentile 3m mdx + FLAVOmega β: 1305, 71,775. 75% Percentile 3m mdx: 3002, 51,825; 75% Percentile 3m mdx + FLAVOmega β 3606, 8955), displaying a considerable presence of myofibers larger than 3000 μm^2^ (Figure 3A). Moreover, AM staining of skeletal muscles showed a downregulation of fibrosis in FLAVOmega β treated mdx mice (*p* < 0.0001) related to untreated age-matched mdx (Figure 3B). Reduced fibrosis deposition and large myofiber area correlated with an improved fatigue resistance, as demonstrated by treadmill outcomes, which show a reduced number of shocks (*p* = 0.0002) (Figure 3D), and a higher latency to fall from the rotating rod (*p* = 0.0001) in FLAVOmega β treated mdx mice (Figure 3C). Based on previous studies, we expected that the amelioration of functional performances may be mediated by an augmented capillary abundance and more organized arteriole distribution [23]. Immunofluorescence staining of FLAVOmega β treated and untreated mdx muscles showed increased percentage of CD31+ cells (*p* < 0.0001) and augmented number of α-SMA+ vessels in 3-month-old mdx mice treated with FLAVOmega β (*p* < 0.0001) compared to age-matched mdx control (Figure 4A). Western blot analysis showed an increased expression of eNOS protein in 3-month-old mdx mice treated with FLAVOmega β (*p* = 0.0282) compared to age-matched mdx control (Figure 4B). Moreover, FLAVOmega β treatment induced a myofiber shift towards oxidative type, likely resulting in functional improvement. Immunofluorescence staining showed an increased expression of type IIa fibers (*p* < 0.0001) and a downregulation of type IIb, IIx and I fibers (IIb: *p* < 0.0001; IIx: *p* = 0.0252; I: *p* = 0.0134) in 3-month-old mdx mice treated with FLAVOmega β (*p* < 0.0001) compared to age-matched mdx control (Figure 4C).

### 3.4. FLAVOmega β Treatment Positively Influences Muscle Regenerative Processes

**Figure 5:**
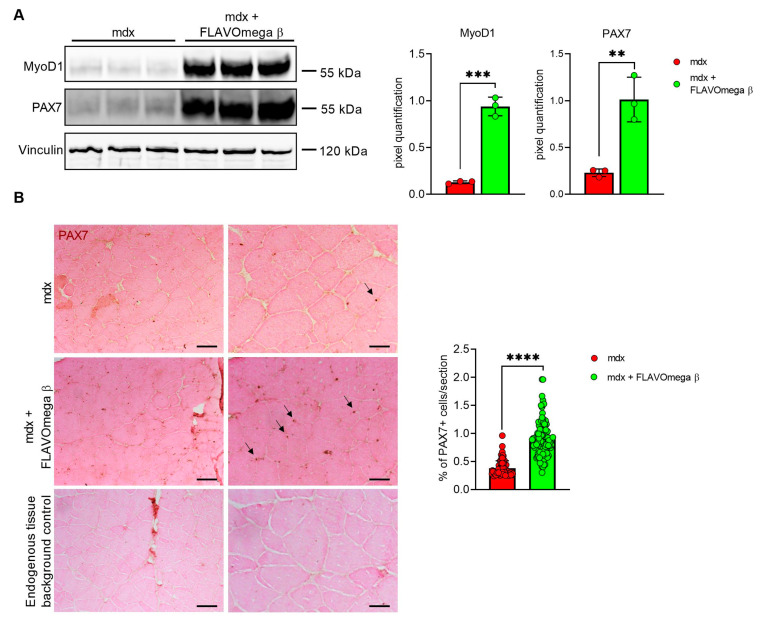
(**A**) Western blot analysis of proteins involved in muscle regeneration process in skeletal muscles of mdx mice treated with FLAVOmega β and untreated mdx mice (*n* = 3 mice/each group). Representative images of PAX7 and MyoD1 blots. In the lateral panel, densitometric analysis of data, expressed as the ratio of the different proteins on vinculin in arbitrary units. *t*-test: ** *p* = 0.0050, *** *p* = 0.0002. (**B**) Representative images of immunohistochemistry staining of mdx mice treated with FLAVOmega β and untreated mdx mice skeletal muscles mice (*n* = 3 mice/each group, *n* = 74 slices/3m mdx, *n* = 129 slices/3m mdx + FLAVOmega β) and endogenous tissue background control. Scale bar left panel: 200 µm; Scale bar right panel: 50 µm. *t*-test: **** *p* < 0.0001.

Based on histological evidence, we investigated some of the proteins involved in muscle regeneration. In Western blot analysis, we found a sustained expression of PAX7 and MyoD1 protein in 3-month-old mdx mice treated with FLAVOmega β (PAX7: *p* = 0.0050; MyoD1: *p* = 0.0002) compared to age-matched mdx mice (Figure 5A). Immunoreactivity of PAX7 staining was also quantified, confirming increased number of PAX7 immunoreactive cells in FLAVOmega β treated mice (*p* < 0.0001) compared to the untreated group (Figure 5B).

### 3.5. Modulation of Metabolism and Downregulation of Inflammatory Pathways in Mdx Mice Treated with FLAVOmega β

**Figure 6:**
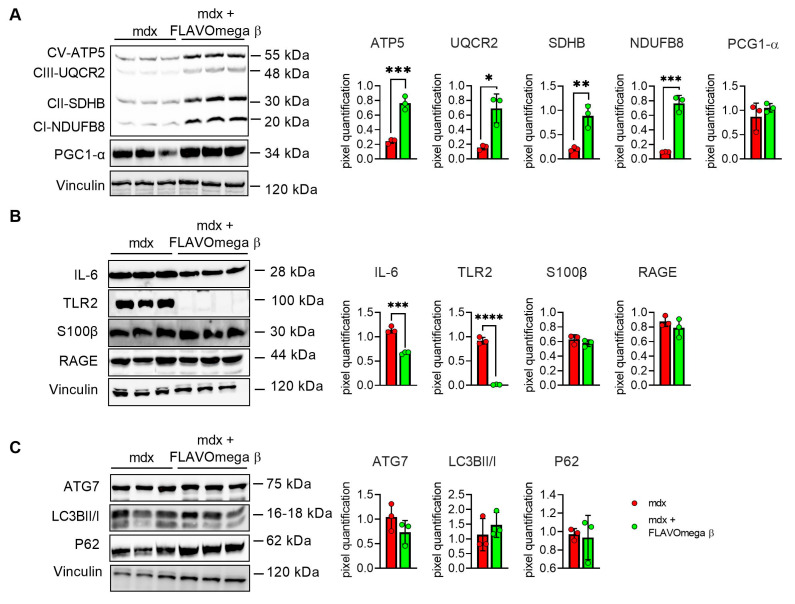
(**A**) Evaluation of OXPHOS complex and PGC1-α expression in skeletal muscles of mdx mice treated with FLAVOmega β and untreated mdx mice (*n* = 3 mice/each group). Representative blots of CV-ATP5A, CIII-UQCRC2, CII-SDHB, CI-NDUFB and PGC1-α. 8. In the lateral panel, densitometric analysis of data, expressed as the ratio of the different proteins on vinculin in arbitrary units. *t*-test: *** *p* = 0.0007, *** *p* = 0.0004, ** *p* = 0.0069, * *p* = 0.0100 (**B**) Evaluation of inflammatory markers in skeletal muscles of mdx mice treated with FLAVOmega β and untreated mdx mice (*n* = 3 mice/each group). Representative blots of IL-6, TLR2, S100 β and RAGE. In the lateral panel, densitometric analysis of data, expressed as the ratio of the different proteins on vinculin in arbitrary units. *t*-test: **** *p* < 0.0001, *** *p* = 0.0003 (**C**) Evaluation of autophagic mediators in skeletal muscles of mdx mice treated with FLAVOmega β and untreated mdx mice (*n* = 3 mice/each group). Representative blots of P62, ATG7 and LC3B. In the lateral panel, densitometric analysis of data, expressed as the ratio of the different proteins on vinculin in arbitrary units. All values are expressed as mean ± SD.

We performed Western blot analysis to investigate metabolism processes and inflammatory pathways. We observed a slight, but not significant, increase of PGC1-α expression, which plays a central role in the regulation of cellular energy metabolism, stimulating mitochondrial biogenesis [24]. Moreover, the analysis confirmed an increase of the OXPHOS complexes in muscles from mdx mice treated with FLAVOmega β, compared with age-matched untreated mice, in line with the abundancy of more oxidative fibers, enriched in mitochondria and capillaries. We detected increased levels of CV-ATP5A (*p* = 0.0007), CIII-UQCRC2 (*p* = 0.0100), CII-SDHB (*p* = 0.0069), CI-NDUFB8 (*p* = 0.0004) after FLAVOmega β treatment (Figure 6A), which also modulated the inflammatory process as demonstrated by the downregulation of IL-6 (*p* = 0.0003) and TLR2 (*p* < 0.0001) in mdx mice treated with FLAVOmega β compared to mdx mice control. Similarly, Western blot analysis on S100-β and RAGE proteins showed a slightly decreased protein level in treated mdx mice compared to the control group (Figure 6B). Finally, we investigated autophagic protein expression, but did not find any relevant modulation of P62, LC3B, and ATG7 proteins after FLAVOmega β treatment (Figure 6C).

### 3.6. Modulation of Dilated Cardiomyopathy Onset in Mdx Mice after FLAVOmega β Administration

**Figure 7:**
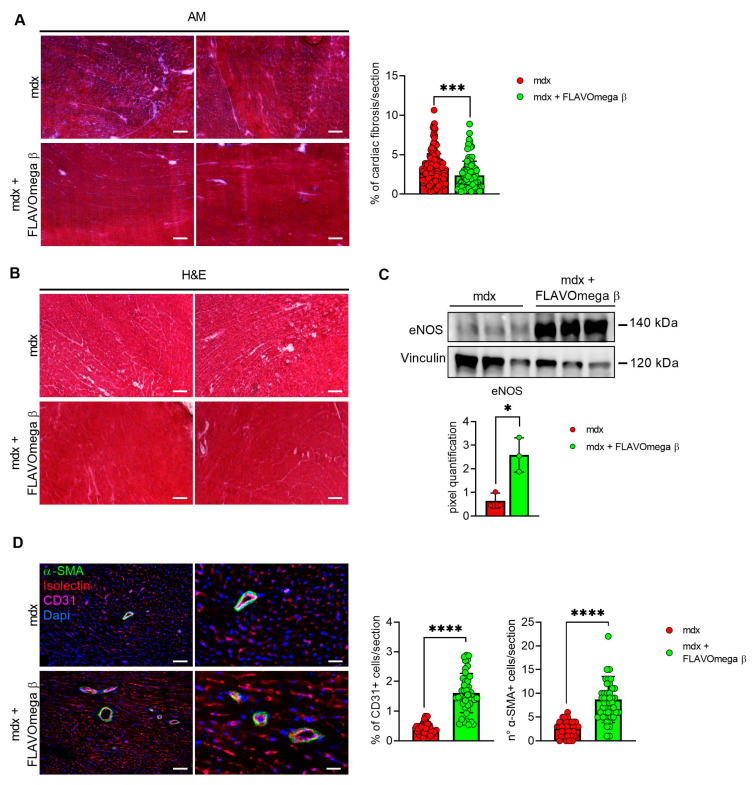
(**A**) Evaluation of fibrosis in cardiac tissues of mdx mice treated with FLAVOmega β and untreated mdx mice. Representative images of AM staining of cardiac tissue of 3-month-old mdx mice (*n* = 3 mice; *n* = 186 slices/3m mdx, *n* = 109 slices/3m mdx + FLAVOmega β). Histogram represents the percentage of fibrotic area per section of mdx mice. Scale bar: 200 µm. *t*-test: *** *p* = 0.0006. (**B**) Representative images of H&E staining of mdx mice treated with FLAVOmega β and untreated mdx mice cardiac tissue. Scale bar: 200 µm. (**C**) Western blot analysis of eNOS protein in cardiac tissue of mdx mice treated with FLAVOmega β and untreated mdx mice (*n* = 3 mice/each group). In the inferior panel, densitometric analysis of data, expressed as the ratio of eNOS on vinculin in arbitrary units. *t*-test: * *p* = 0.0136. (**D**) Immunofluorescence analysis of CD31 (violet), isolectin (red) and α-SMA (green) in cardiac tissue of mdx mice treated with FLAVOmega β and untreated mdx mice (*n* = 3 mice/each group, *n* = 9 sections/3m mdx, *n* = 9/3m mdx + FLAVOmega β). Nuclei were counterstained with DAPI (blue). Scale bar left panel: 100 µm; scale bar right panel: 25 µm. *t*-test: **** *p* < 0.0001. All values are expressed as mean ± SD.

**Figure 8:**
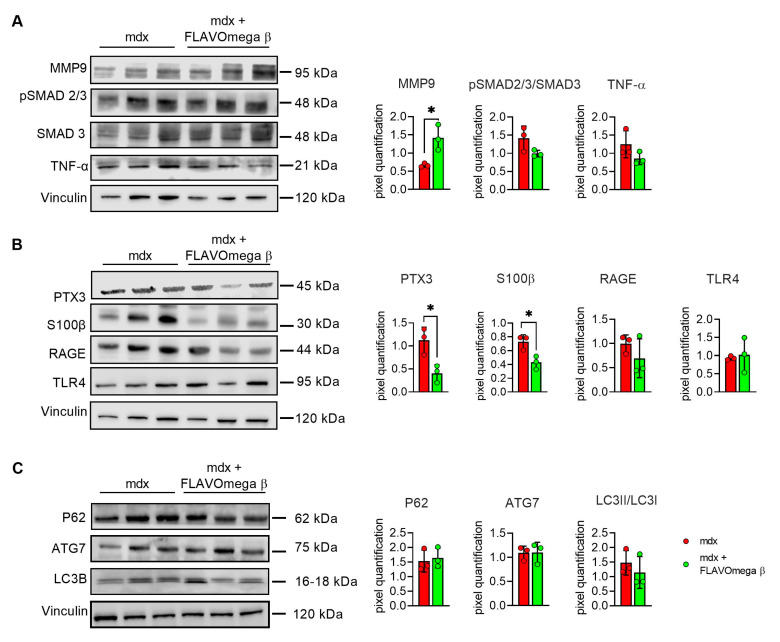
(**A**–**C**) Western blot analysis of fibrotic, inflammatory and autophagic pathways in cardiac tissue of mdx mice treated with FLAVOmega β and untreated mdx mice (*n* = 3 mice/each group). (**A**) Representative blots of TNF-α, MMP9, pSMAD2-3 and SMAD3. In the lateral panel, densitometric analysis of data, expressed as the ratio of the different proteins on vinculin in arbitrary units. *t*-test: * *p* = 0.0207. (**B**) Representative blots of PTX3 mechanism and its alarmins: RAGE, S100β and TLR4. In the lateral panel, densitometric analysis of data, expressed as the ratio of the different proteins on vinculin in arbitrary units. *t*-test: * *p* = 0.0222, * *p* = 0.0214. (**C**) Representative blots of LC3B, P62 and ATG7. In the lateral panel, densitometric analysis of data, expressed as the ratio of the different proteins on vinculin in arbitrary units. All values are expressed as mean ± SD.

We evaluated cardiac morphology and fibrotic process through histological analysis. As observed in skeletal muscles, AM staining showed a downregulation of fibrosis in 3-month-old mdx mice treated with FLAVOmega β (*p* = 0.0006) related to untreated age-matched mdx (Figure 7A) also in cardiac tissues. Vascularization was also modulated after FLAVOmega β treatment. Western blot analysis showed an increased expression of eNOS in cardiac tissue of mdx mice treated with FLAVOmega β (*p* = 0.0136) compared to age-matched mdx control (Figure 7C), with an increased number of CD31+ positive cells in treated mdx mice (*p* < 0.0001) (Figure 7D). Through Western blot analysis, we found an upregulation of MMP9 in 3-month-old mdx mice treated with FLAVOmega β (*p* = 0.0207) related to untreated age-matched mdx mice (Figure 8A). MMP-9 enzymatically cleaves numerous extracellular matrix substrates and cytokines/chemokines including TNFα and pSMAD2-3 to facilitate cardiac remodeling [25]. Thus, MMP-9 is capable of propagating cardiac inflammatory signaling that is both necessary for cardiac wound healing and potentially deleterious in sustenance of chronic inflammation. Among cardiac inflammatory mediators, we previously described the role of alarmins in prolonging inflammation in DMD cardiomyopathy influencing the expression of PTX3. One possible mechanism through which MMP-9 may blunt cardiac inflammation could be by reducing S100-β alarmin and PTX3 protein levels. In FLAVOmega β treated muscles, we observed a low expression of TNF-α and pSMAD2-3 and the downregulation of S100-β alarmin and PTX3 expression, usually highly expressed in dystrophic cardiomyopathy [26], in 3-month-old mdx mice treated with FLAVOmega β (S100-β: *p* = 0.0214; PTX3: *p* = 0.0222) compared to age-matched mdx control (Figure 8B). All these data suggest an anti-inflammatory effect of FLAVOmega β in dystrophic cardiac remodeling. Autophagy is a process essential for heart maintenance and adaptation, and it is usually highly sensitive to cardiac stress and failure. Although proteins associated to autophagic flux did not show major changes, FLAVOmega β treated mdx mice displayed slight P62 deposition and a considerable LC3B flux, that may suggest a modulation of the autophagic machinery (Figure 8C).

## 4. Discussion

Food supplements are concentrated sources of nutrients or other substances with a physiological effect to support the normal diet [27]. All types of supplements have the role of balancing the physiological conditions and many of these can be used as adjuvants of drug therapies. DMD patients, lacking dystrophin expression, suffer from muscle weakness, loss of ambulation, cardio-respiratory complications, metabolic and gastrointestinal problems [28]. The molecular phenotype in dystrophic patients consist in persistent disturbance of muscle homeostasis, myofiber necrosis, fibrosis deposition, influx of inflammatory cells, and severe mitochondrial dysfunction [29]. Indeed, nutraceutical therapies targeting inflammation and oxidative stress may be helpful to counteract, or at least delay, DMD pathology by preserving physiological muscle conditions and functional performance for longer time. Our group and others have identified the beneficial role of flavonoids and omega-3 fatty acids in dystrophic skeletal muscle metabolism [7]. However, the molecular mechanisms that mediate effects of these compounds in dystrophic muscle are not well known. In the present study, we identified a new food supplement formulation named FLAVOmega β, which induces a generalized improvement of the dystrophic phenotype by modulating inflammation and oxidative stress. Our preliminary in vitro studies demonstrated that FLAVOmega β is able to promote myogenic differentiation of C2C12 cells and exert a moderate ROS scavenging activity in condition of normal and high oxidative stress, in absence of evident cytotoxic effects. Oxidative stress has a relevant role in the occurrence of the dystrophic phenotype and causes free-radical induced necrosis in myoblasts, therefore interfering with their subsequent differentiation toward mature and functional fibers [30]. To evaluate the effects of the supplement in vivo, we treated mdx mice of 3 months of age, daily, for one month—at this stage of life the dystrophic features become pronounced with occurrence of necrotic or regenerating myofibers, infiltrates of inflammatory cells and high levels of pro-inflammatory cytokines. Overall, muscle cross sectional area (CSA) significantly increased in FLAVOmega β treated mice, suggesting a successful progression of regenerating fibers towards mature stages and the protection from atrophic conditions with maintenance of muscle mass [31]. Injured fibers in FLAVOmega β treated muscles were replaced by a sustained muscle regeneration that may depend on a declined rate of degenerative/regenerative myofiber cycles [32].

In fact, we showed an increased MyoD1 and PAX7 expression and higher number of satellite cells compared to untreated mdx mice, indicating a less detrimental muscle environment for myogenic cell proliferation and proper differentiation after injury.

Moreover, muscles of FLAVOmega β-treated mice showed few cells infiltrate areas, thin interstitial connective tissue between fibers, and restricted fibrosis, suggesting a modulation of the fibrotic and inflammatory trend typical of the morphological patterns of dystrophic muscles [33]. We also found a significant downregulation of IL-6 and TLR-2 [34]. These results confirmed the expected anti-inflammatory properties of the components of our mixture and explained the overall improvement observed in the muscular performance of treated mice. Indeed, the above-mentioned histological findings were accompanied by an improved resistance to fatigue, balance and motor coordination, as demonstrated by Rotarod and treadmill analyses.

The ameliorated exercise performance could also be explained by a tuning of the muscle metabolism toward an oxidative one. FLAVOmega β supplementation produced a higher percentage of oxidative myofibers, in particular the so called “fast oxidative” type IIa ones, that significantly exceeded the number of glycolytic type IIb fibers. Although we did not find evidence for enhanced mitochondrial biogenesis, this oxidative shift was supported by the augmented expression of mitochondrial oxidative phosphorylation complexes (OXPHOS) in muscle of treated mice [35,36,37]. Flavonoids and omega-3 fatty acids might impact oxidative metabolism by increasing mitochondrial activity and metabolic gene expression through activation of lipid-sensing transcription factors [38,39,40]. Improved oxidative potential likely relies on the rescue of mitochondrial dysfunctions, which originates since the early onset of DMD pathology, worsens with pathology progression [40], and is accompanied by ROS and inflammatory cytokines production. Interestingly, we also demonstrated abundance of CD31+ cells in FLAVOmega β-treated muscles with upregulation of eNOS, which is an enzyme with protective role on the vasculature branches and tone [41]. These findings correlate with a remodeling of the vascular apparatus of the muscle that may result from an attempt to support the high metabolic demand of oxidative fibers.

Dilated cardiomyopathy is the major cause of death in patients with DMD. Previous studies demonstrated a protective effect of flavonoids and omega-3 fatty acids against heart disease complications [42,43]. Experiments conducted on cardiac tissue of mdx mice treated with FLAVOmega β showed a moderately preserved cardiac tissue structure accompanied by the lack of inflammatory infiltrate population, the decrease in fibrotic tissue and the upregulation of vascular markers. These cardiometabolic benefits might be related to improved endothelial production of eNOS, as observed in skeletal muscle [44], and to a modulation of PTX3-related pathways, which are known to be predictive of myocardial damage and fibrosis in mdx mice. However, due to its late onset (starting from 8 month of age), the effective role of FLAVOmega β in the modulation of the dilated cardiomyopathy phenotype is yet to be established via additional in vivo studies on older mice [26].

The complexity of these cardiac and muscular metabolic responses highlights the rapid advances in nutritional science and the continued need to generate robust empirical evidence on the mechanistic and clinical effects of specific foods. Although the exact mechanisms that govern the broad metabolic effects of flavonoids and omega-3 fatty acids in dystrophic muscles are not yet understood, our results provide new insights into the health benefits brought by these supplements to contribute in the maintenance of muscle function in DMD and constitute a preliminary indication for translation to clinical setting.

## Figures and Tables

**Table 1:** Specific composition of formulations used for in vitro assays.

	µM	FLAVO α	FLAVO β	FLAVO γ	FLAVOmega α	FLAVOmega β	FLAVOmega γ
Powder phase	Curcumin	1	5	10	1	1	1
	Coenzyme Q10	0.085	0.425	0.850	0.085	0.085	0.085
	Baicalein	0.082	0.410	0.820	0.082	0.082	0.082
	Epicatechins	0.088	0.444	0.888	0.088	0.088	0.088
	Aloe vera	0.023	0.115	0.230	0.023	0.023	0.023
Oil phase	DHA				1.4	7	14
	EPA				0.69	3.45	6.9

## Data Availability

The data presented in this study are available in the article.
